# Artificial intelligence-based decision support for axial spondyloarthritis integrating inflammatory and structural MRI information

**DOI:** 10.3389/fimmu.2026.1950510

**Published:** 2026-10-19

**Authors:** Yunseo Kim, Seulkee Lee, Seonyoung Kang, Jaejoon Lee, Myung Jin Chung, Ji Hyun Lee, Hakje Yoo, Hoon-Suk Cha

**Affiliations:** 1 Medical Artificial Intelligence (AI) Research Center, Research Institute for Future Medicine, Samsung Medical Center, Seoul, Republic of Korea; 2 Department of Digital Health, Samsung Advanced Institute for Health Sciences & Technology (SAIHST), Sungkyunkwan University, Seoul, Republic of Korea; 3 Department of Medicine, Samsung Medical Center, Sungkyunkwan University School of Medicine, Seoul, Republic of Korea; 4 Department of Radiology, Samsung Medical Center, Sungkyunkwan University School of Medicine, Seoul, Republic of Korea; 5 Department of Data Convergence and Future Medicine, Sungkyunkwan University School of Medicine, Seoul, Republic of Korea; 6 Department of Biomedical Software Engineering, The Catholic University of Korea, Bucheon, Republic of Korea

**Keywords:** artificial intelligence, axial spondyloarthritis, machine learning, MRI, sacroiliitis

## Abstract

**Background:**

Axial spondyloarthritis (axSpA) assessment on MRI is complicated by interpretation difficulty and by clinical variables that may be incomplete at the time of imaging. We aimed to develop an image-only deep learning framework to approximate the clinical assessment of axSpA from sacroiliac joint (SIJ) MRI, integrating fat-suppressed T1-weighted (FS T1) and short tau inversion recovery (STIR) sequences to capture structural and inflammatory information without clinical variables.

**Methods:**

This retrospective study included patients with chronic back pain who underwent SIJ MRI from January 2010 to December 2021. The treating rheumatologist’s clinical diagnosis served as the reference standard. A two-stage framework integrated FS T1 and STIR features with Stage 1-derived binary bone marrow edema (BME) predictions to generate an axSpA probability. Performance in a test set was compared with the Assessment of SpondyloArthritis International Society (ASAS) definition of a “positive MRI” and the ASAS classification criteria using exact McNemar tests; discordant cases were analyzed separately.

**Results:**

Among 291 patients (163 axSpA, 128 non-axSpA), the model achieved an AUROC of 0.9412, accuracy of 86.0%, sensitivity of 87.5%, and specificity of 84.0% in the test set (n=57). No significant paired differences in accuracy, sensitivity, or specificity were detected between the model and either ASAS comparator. In an exploratory analysis of 10 cases discordant between ASAS “positive MRI” assessment and clinical diagnosis, the model correctly classified 7 (70.0%).

**Conclusion:**

The model showed similar performance to the ASAS-based comparators in the test set without clinical variables, suggesting potential value as an image-based decision-support tool. External multicenter validation is needed to confirm generalizability.

## 1 Introduction

Axial spondyloarthritis (axSpA) is a chronic inflammatory disease that primarily involves the sacroiliac joint (SIJ) (1, 2). Because magnetic resonance imaging (MRI) can detect sacroiliitis early by identifying bone marrow edema (BME), the Assessment of SpondyloArthritis International Society (ASAS) has defined sacroiliitis consistent with axSpA, termed “positive MRI,” based on subchondral BME on oblique coronal short tau inversion recovery (STIR) sequence (3, 4). However, interpreting SIJ magnetic resonance imaging (MRI) remains labor-intensive, requires substantial expertise, and is subject to considerable variability among readers (5, 6). As artificial intelligence (AI) has recently shown remarkable performance in medical imaging (7–9), it has been applied to SIJ imaging to overcome these interpretive limitations (10).

However, what clinicians ultimately need to determine is not whether a particular imaging finding is present, but whether a patient has axSpA, and in this respect existing image-based approaches have limitations. The “positive MRI” that has served as the prediction target in most prior work is based solely on BME, which can yield false-positive findings under conditions of mechanical stress, as observed in athletes and postpartum women (11–13), and may be absent in patients with established but currently inactive disease, resulting in false-negative results (14). Although incorporating structural changes alongside BME has been reported to improve diagnostic accuracy for axSpA, consensus on which specific structural lesions should be included in classification criteria has not yet been reached (15). Previous AI studies in spondyloarthritis have included radiographic severity assessment in ankylosing spondylitis (16), while machine-learning studies targeting axSpA diagnosis relied on clinical or electronic health record variables (17, 18), and a recent multicenter study combined clinical risk factors with SIJ MRI (19). However, information such as HLA-B27 status may be unavailable at the time of initial imaging, particularly in primary care settings.

In this study, we developed a deep learning framework for approximating the clinical assessment of axSpA using SIJ MRI alone, incorporating fat-suppressed T1-weighted (FS T1) and STIR sequences as complementary imaging inputs. We further explored how this image-only framework performed relative to the ASAS definition of a “positive MRI” and the ASAS classification criteria, used here solely as research comparators, and whether it could serve as an image-based decision-support tool when clinical information is incomplete.

## 2 Materials and methods

### 2.1 Patients and study design

This retrospective study initially screened 302 adult patients (≥18 years) with chronic back pain who underwent SIJ MRI at the Department of Rheumatology, Samsung Medical Center, between January 2010 and December 2021. Chronic back pain was defined as back pain persisting for at least three months. MRI was obtained as part of clinical care rather than under a dedicated study protocol, and all patients underwent oblique coronal STIR and FS T1 sequences according to the institutional standard SIJ MRI protocol. Patients were excluded if required MRI sequences or annotation labels were missing, if duplicate records were identified, if SIJ assessment was infeasible because of metallic artifacts, or if the required clinical information was unavailable.

A total of 302 patients were assessed for eligibility, of whom 11 were excluded: 4 because of incomplete MRI sequences, 4 because of missing annotation labels, 1 duplicate patient, 1 because of a metallic artifact preventing evaluation, and 1 because of missing clinical information. The remaining 291 patients were included in the final analysis and classified according to the clinical diagnosis made by the treating rheumatologist: 163 had axSpA and 128 did not have axSpA (**Supplementary Figure S1**).

### 2.2 MRI acquisition and annotation

All MRI examinations were acquired at 3.0 T using three scanner systems from two vendors. The institutional SIJ MRI protocol included two-dimensional oblique coronal FS T1 and STIR sequences. For Dixon acquisitions, the water-only reconstruction was used as the FS T1 image. Slice thickness was 4.0 mm with a slice spacing of 4.4 mm, corresponding to a 0.4-mm interslice gap, in all examinations. Detailed scanner models and acquisition parameters are provided in **Supplementary Table S3**.

Bounding boxes for the SIJs were annotated independently by a rheumatologist (SL) and a radiologist (MJC), and BME was assessed independently by a rheumatologist (SL) and a musculoskeletal radiologist (JHL). Both assessments were performed using de-identified, coded MRI examinations, with the readers blinded to patient identity, clinical information, and clinical diagnosis. BME was rated according to the ASAS definition of active sacroiliitis as subchondral BME at characteristic locations on STIR sequences, present either as (i) two or more lesions on a single slice or (ii) a single lesion on at least two consecutive slices (3). Interreader agreement was assessed using Cohen’s kappa. Disagreements were resolved by consensus, and the consensus labels were used for model training and evaluation.

### 2.3 ASAS classification criteria assessment

The ASAS classification criteria were retrospectively applied using available clinical, laboratory, and imaging information obtained through chart review. At our rheumatology clinic, clinical features relevant to the ASAS classification criteria are routinely documented as part of the initial evaluation of patients with suspected axSpA. Available longitudinal medical records were also reviewed, and relevant features documented during subsequent follow-up were incorporated when available. The imaging and clinical arms of the ASAS classification criteria were then applied using these retrospectively collected data. The consensus BME assessment described above was also used to determine whether each MRI met the ASAS definition of a “positive MRI” for the research comparator analysis.

### 2.4 Reference standard

The reference standard for model training and evaluation was the final clinical diagnosis documented by the treating board-certified rheumatologist, ascertained through longitudinal review of each patient’s documented clinical course. The treating rheumatologist’s diagnosis reflected an integrated assessment of clinical symptoms, laboratory findings, imaging findings, and subsequent disease course. The reference standard was not defined at a specific clinical time point or according to a fixed temporal relationship with MRI acquisition. In this study, the ASAS definition of a “positive MRI” and the ASAS classification criteria described above were not used as reference standards but were evaluated separately as research comparators.

### 2.5 MRI preprocessing

Each DICOM slice was min–max normalized to an 8-bit range, with the DICOM window center and width applied beforehand for patient-level classification. Histogram matching was performed using fixed mid-joint reference images selected exclusively from the training set; no validation- or test-set images were used for reference selection. CLAHE was applied using a clip limit of 0.01 and a 64 × 64-pixel kernel for SIJ localization and an 8 × 8 tile grid with clip limits of 2.0 and 4.0 for BME and patient-level classification, respectively (**Supplementary Figure S2**).

### 2.6 Two-stage framework for SIJ detection and axSpA classification

The proposed framework is a sequential two-stage architecture for patient-level classification of axSpA from SIJ MRI (**Figure 1**). In the first stage, the SIJs were localized and slice-level BME was predicted from STIR images. A YOLOv12-based detector (20) localized the left and right SIJs, and the detected regions of interest were analyzed with a ConvNeXt-based classifier (21) to estimate the presence of BME on each side. A slice was defined as BME-positive if either joint showed edema. These binary slice-level BME predictions served as explicit inflammatory cues for patient-level classification.

**Figure 1:**
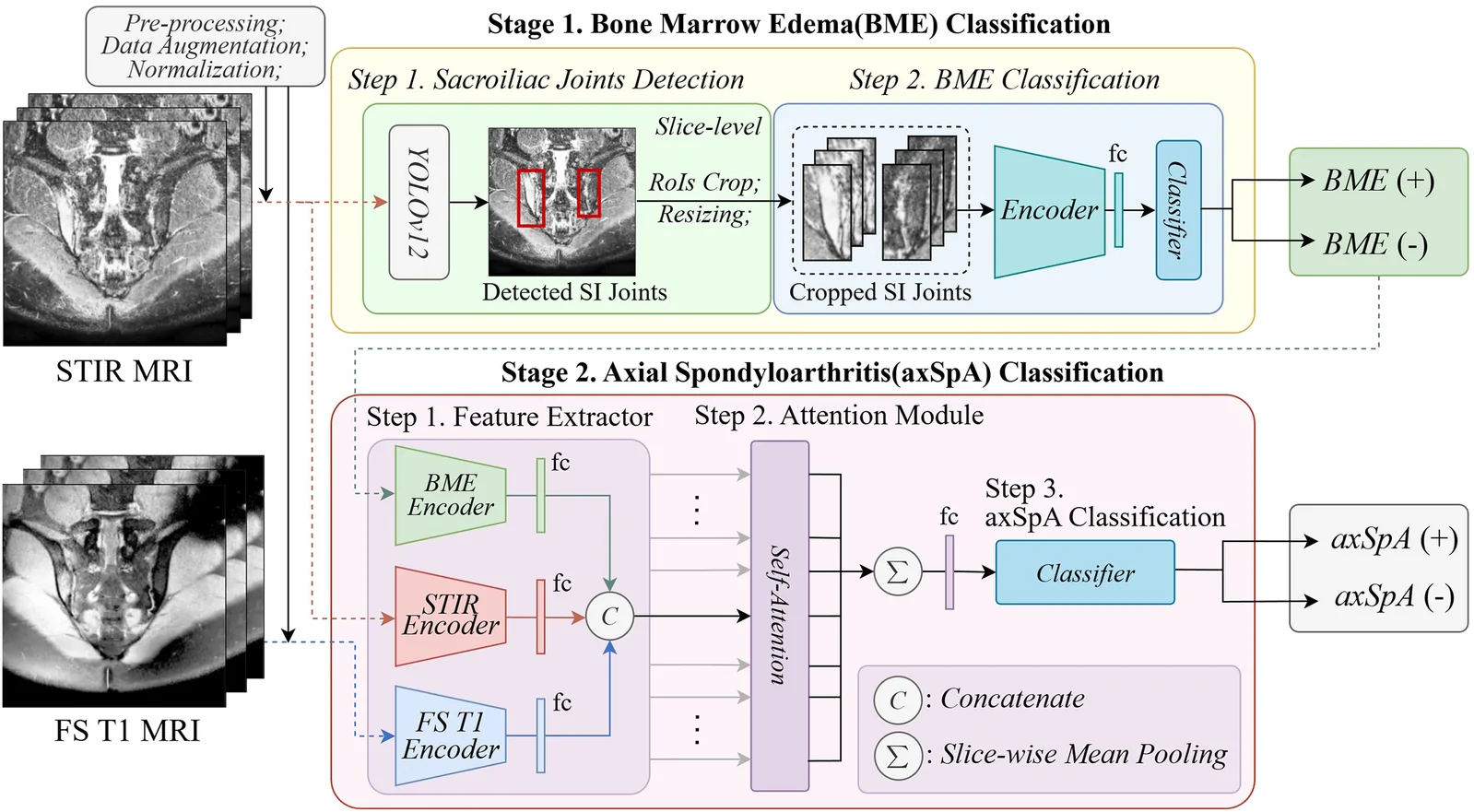
Overview of the two-stage framework for patient-level classification of axial spondyloarthritis (axSpA) from sacroiliac joint (SIJ) MRI. Stage 1 localizes the SIJs and generates slice-level bone marrow edema (BME) predictions from short tau inversion recovery (STIR) images, whereas Stage 2 integrates fat-suppressed T1-weighted (FS T1), STIR, and BME information using attention-based aggregation.

In the second stage, axSpA was classified at the patient level. For each patient, features were extracted from FS T1 and STIR images using EfficientNet-based encoders (22), while the binary slice-level BME predictions generated in the first stage were processed in parallel through a multilayer perceptron. The resulting modality-specific features were concatenated per slice and integrated into a patient-level representation using an attention-based aggregation mechanism (23), which assigned higher weights to diagnostically informative slices. The aggregated representation was then passed through a fully connected layer to produce the probability of axSpA.

### 2.7 Model training

Patients were divided at the patient level into mutually exclusive training (n=187), validation (n=47), and hold-out test (n=57) sets. The same patient assignments were maintained across SIJ localization, BME classification, and patient-level axSpA classification, and all slices and regions of interest derived from each patient remained within the assigned subset. The training set was used for model fitting, the validation set for checkpoint selection and early stopping, and the hold-out test set for final evaluation only.

For SIJ localization, checkpoint selection and early stopping followed the default Ultralytics validation fitness criterion. For BME and axSpA classification, the checkpoint with the lowest validation loss was selected. Model architectures and hyperparameters were selected empirically during development using the training and validation sets, without systematic or automated optimization. Once determined, the final configurations were fixed before evaluation on the hold-out test set. A classification threshold of 0.5 was prespecified for both BME and axSpA classification. Detailed sample distributions and final training configurations are provided in **Supplementary Tables S1** and **S2**.

### 2.8 Evaluation and comparisons

The proposed model was evaluated on a separate hold-out test set. SIJ localization was evaluated using mean intersection-over-union and mean average precision, and axSpA classification was evaluated using the area under the receiver operating characteristic curve (AUROC), accuracy, sensitivity, and specificity. To assess the contributions of FS T1 and STIR, we performed an ablation analysis across three input combinations (FS T1+STIR+BME, FS T1+BME, and STIR+BME). The proposed model was further compared with the ASAS “positive MRI” definition and the ASAS classification criteria, and BME-clinical discordant cases were analyzed separately.

### 2.9 Statistical analysis

Baseline characteristics are presented as mean (standard deviation) for continuous variables and as number (percentage) for categorical variables. Differences between the axSpA and non-axSpA groups were assessed using the t-test or Mann–Whitney U test for continuous variables and the chi-squared test or Fisher’s exact test for categorical variables. Pairwise comparisons of accuracy, sensitivity, and specificity between the proposed model and each ASAS-based classification were performed using exact McNemar tests. These comparisons were not designed for formal non-inferiority testing and should be interpreted as exploratory and hypothesis-generating. All tests were two-sided, p<0.05 was considered statistically significant, and 95% confidence intervals for performance metrics were calculated using bootstrap resampling with 2,000 iterations.

## 3 Results

### 3.1 Dataset characteristics

Among the 291 patients (163 axSpA, 128 non-axSpA), age (35.69 ± 12.57 vs. 38.19 ± 12.11 years, p=0.088) and sex distribution (male, 62.0% vs. 53.9%, p=0.206) did not differ significantly (**Table 1**). Patients with axSpA had a longer symptom duration (3.88 ± 4.96 vs. 2.22 ± 3.02 years, p=0.003) and higher inflammatory markers (ESR, 32.91 ± 33.20 vs. 15.84 ± 17.59 mm/h; CRP, 1.33 ± 2.42 vs. 0.34 ± 1.08 mg/dL; both p<0.001). HLA-B27 positivity (92.0% vs. 40.6%, p<0.001), inflammatory back pain (75.5% vs. 43.8%, p<0.001), and uveitis (22.1% vs. 10.2%, p=0.011) were also more frequent in the axSpA group. These findings were consistent with the established clinical and laboratory profile of axSpA, including frequent HLA-B27 positivity and elevated inflammatory markers. Notably, HLA-B27 positivity and spondyloarthritis features were also observed in a substantial proportion of the non-axSpA group, reflecting the clinically relevant differential-diagnosis setting of this cohort. Interreader agreement for ASAS-defined sacroiliitis was high (Cohen’s kappa 0.876, 95% CI 0.771–0.981).

**Table 1:** Baseline patient demographic, clinical, and laboratory characteristics of the study cohort (n=291), stratified by axSpA and non-axSpA patients.

Characteristics	Total (*n* = 291)	axSpA (*n* = 163)	non-axSpA (*n* = 128)	*p-*value
Age, mean (SD), year	36.8 (12.4)	35.7 (12.6)	38.2 (12.1)	0.09
Male sex, n (%)	170 (58.4)	101 (62.0)	69 (53.9)	0.21
Duration of back pain, mean (SD), years	3.2 (4.3)	3.9 (5.0)	2.2 (3.0)	0.003
Inflammatory back pain, n (%)	179 (61.5)	123 (75.5)	56 (43.8)	< 0.001
Peripheral arthritis, n (%)	67 (23.0)	44 (27.0)	23 (18.0)	0.09
Enthesitis, n (%)	32 (11.0)	21 (12.9)	11 (8.6)	0.33
Dactylitis, n (%)	3 (1.0)	2 (1.2)	1 (0.8)	> 0.99
IBD, n (%)	5 (1.7)	2 (1.2)	3 (2.3)	0.79
Uveitis, n (%)	49 (16.8)	36 (22.1)	13 (10.2)	0.01
Psoriasis, n (%)	6 (2.1)	1 (0.6)	5 (3.9)	0.12
HLA-B27, n (%)				< 0.001
Negative	82 (28.2)	9 (5.5)	73 (57.0)	
Positive	202 (69.4)	150 (92.0)	52 (40.6)	
Unknown	7 (2.4)	4 (2.5)	3 (2.3)	
ESR, mean (SD), mm/h	25.3 (28.6)	32.9 (33.2)	15.8 (17.6)	< 0.001
CRP, mean (SD), mg/dL	0.9 (2.0)	1.3 (2.4)	0.3 (1.1)	< 0.001
ASAS axSpA classification criteria, n (%)	171 (58.8)	157 (96.3)	14 (10.9)	< 0.001
Non-radiographic axSpA, n (%)	NA	69 (42.3)	NA	NA

Values are presented as mean (SD) or n (%), unless otherwise indicated. ESR, erythrocyte sedimentation rate; CRP, C-reactive protein; IBD, inflammatory bowel disease; ASAS, assessment of spondyloarthritis international society; axSpA, axial spondyloarthritis; SD, standard deviation; NA, not applicable.

### 3.2 Model performance

In the first stage, the YOLOv12-based SIJ localization model achieved a mean average precision of 0.9852 at an IoU threshold of 0.5 (**Supplementary Figure S3**), and the ConvNeXt-based BME classification model achieved an AUROC of 0.9186 with a sensitivity of 0.8938 at the region-of-interest level (**Table 2**; **Figure 2A**). On Grad-CAM analysis, the classifier’s activations consistently focused on the periarticular regions adjacent to the SIJs (**Figure 3**).

**Table 2:** Performance of Stage 1 tasks: SIJ detection and BME classification.

Task	Metric	Value
SIJs Detection	mAP@0.5	0.9852
	mAP@0.75	0.8505
	Mean IoU (95% CI)	0.8203 (0.8118-0.8282)
BME Classification	Accuracy (95% CI)	0.8244 (0.7985-0.8481)
	Sensitivity (95% CI)	0.8938 (0.8540-0.9292)
	Specificity (95% CI)	0.8020 (0.7721-0.8305)
	AUROC (95% CI)	0.9186 (0.8966-0.9389)

SIJs: Sacroiliac joints; mAP: mean average precision; IoU: Intersection over union; BME: bone marrow edema; AUROC: area under the receiver operating characteristics curve. Localization performance is reported for SIJ detection. BME classification was evaluated on 928 SIJ ROIs (positives = 226, negatives = 702).

**Figure 2:**
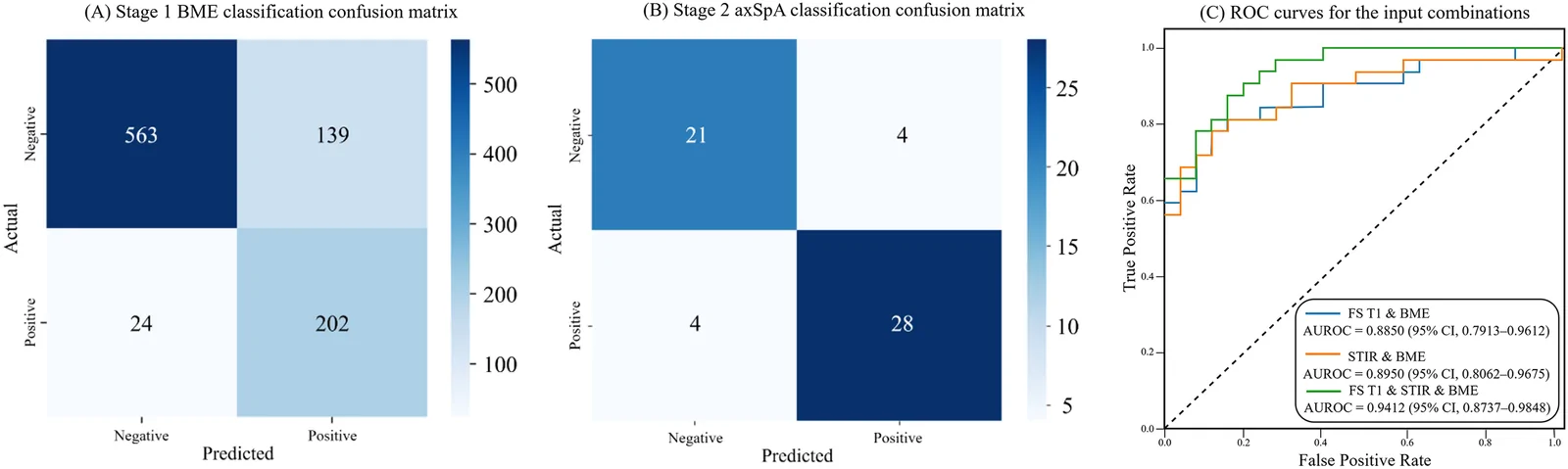
Classification performance of the two-stage framework. **(A)** Confusion matrix for Stage 1 bone marrow edema (BME) classification. **(B)** Confusion matrix for patient-level axial spondyloarthritis (axSpA) classification using the full model. **(C)** Receiver operating characteristic curves for the three input combinations evaluated in the hold-out test set.

**Figure 3:**
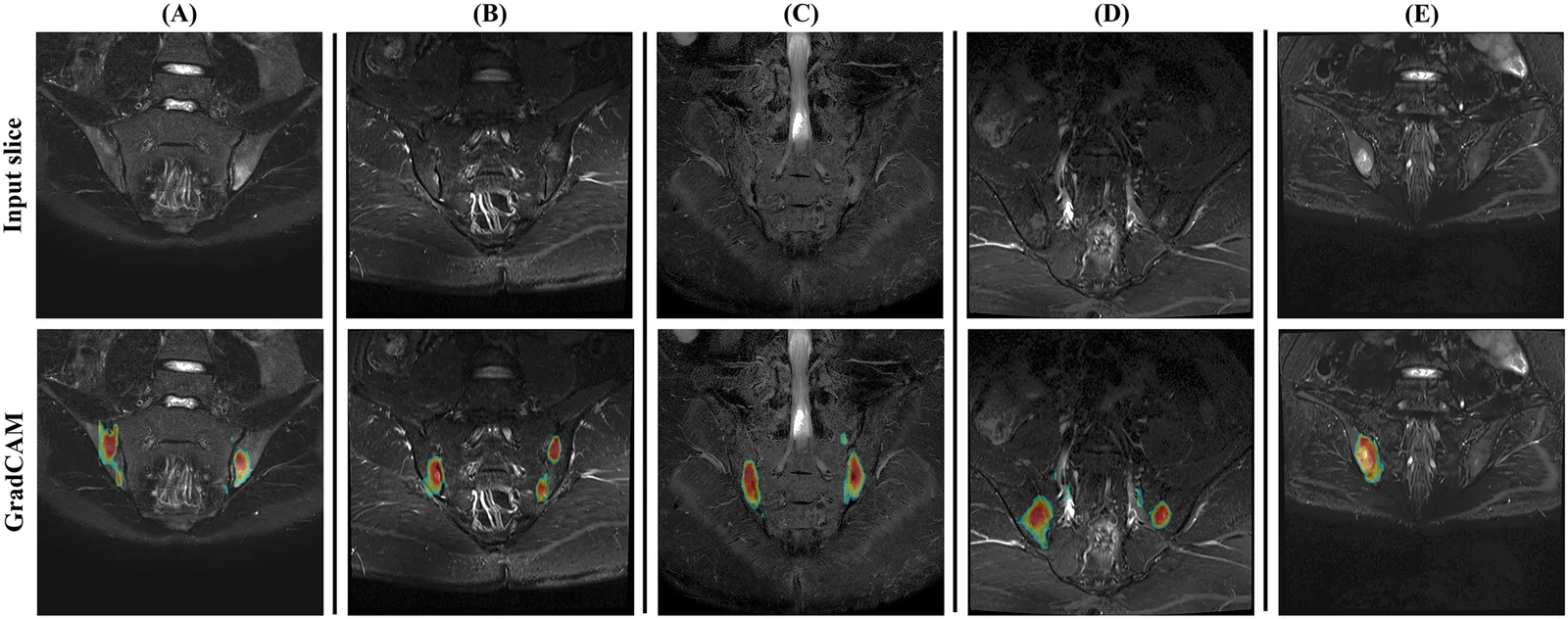
Representative Grad-CAM maps from the Stage 1 bone marrow edema (BME) classifier on short tau inversion recovery (STIR) MRI. The top row shows input slices **(A–E)**, and the bottom row shows the corresponding Grad-CAM maps, with activations predominantly localized to the periarticular regions of the sacroiliac joints.

In the second stage, the full model correctly classified 49 of 57 patients, including 28 of 32 patients with axSpA and 21 of 25 patients without axSpA (**Figure 2B**). It achieved an AUROC of 0.9412 and showed numerically higher AUROC and sensitivity than the reduced-modality models (FS T1+BME and STIR+BME) (**Table 3**; **Figure 2C**). On slice-level attention analysis, axSpA-positive patients showed higher weights for the central slices (indices 4–8), where the SIJs are most clearly visualized, whereas patients without axSpA showed a relatively uniform distribution across slices (**Figure 4**).

**Table 3:** Patient-level axSpA classification performance on the test set (n=57) for three input modality combinations.

Input	AUROC (95% CI)	Accuracy (95% CI)	Sensitivity (95% CI)	Specificity (95% CI)
(a)	0.8850 (0.7913-0.9612)	0.8070 (0.7018-0.9123)	0.7500 (0.5938-0.9062)	0.8800 (0.7600-1.0000)
(b)	0.8950 (0.8062-0.9675)	0.8421 (0.7368-0.9298)	0.8125 (0.6562-0.9375)	0.8800 (0.7600-1.0000)
(c)	0.9412 (0.8737-0.9848)	0.8596 (0.7719-0.9298)	0.8750 (0.7436-0.9697)	0.8400 (0.6842-0.9615)

(a) T1 + BME; (b) STIR + BME; (c) T1 + STIR + BME. The full model achieved the highest AUROC and sensitivity.

**Figure 4:**
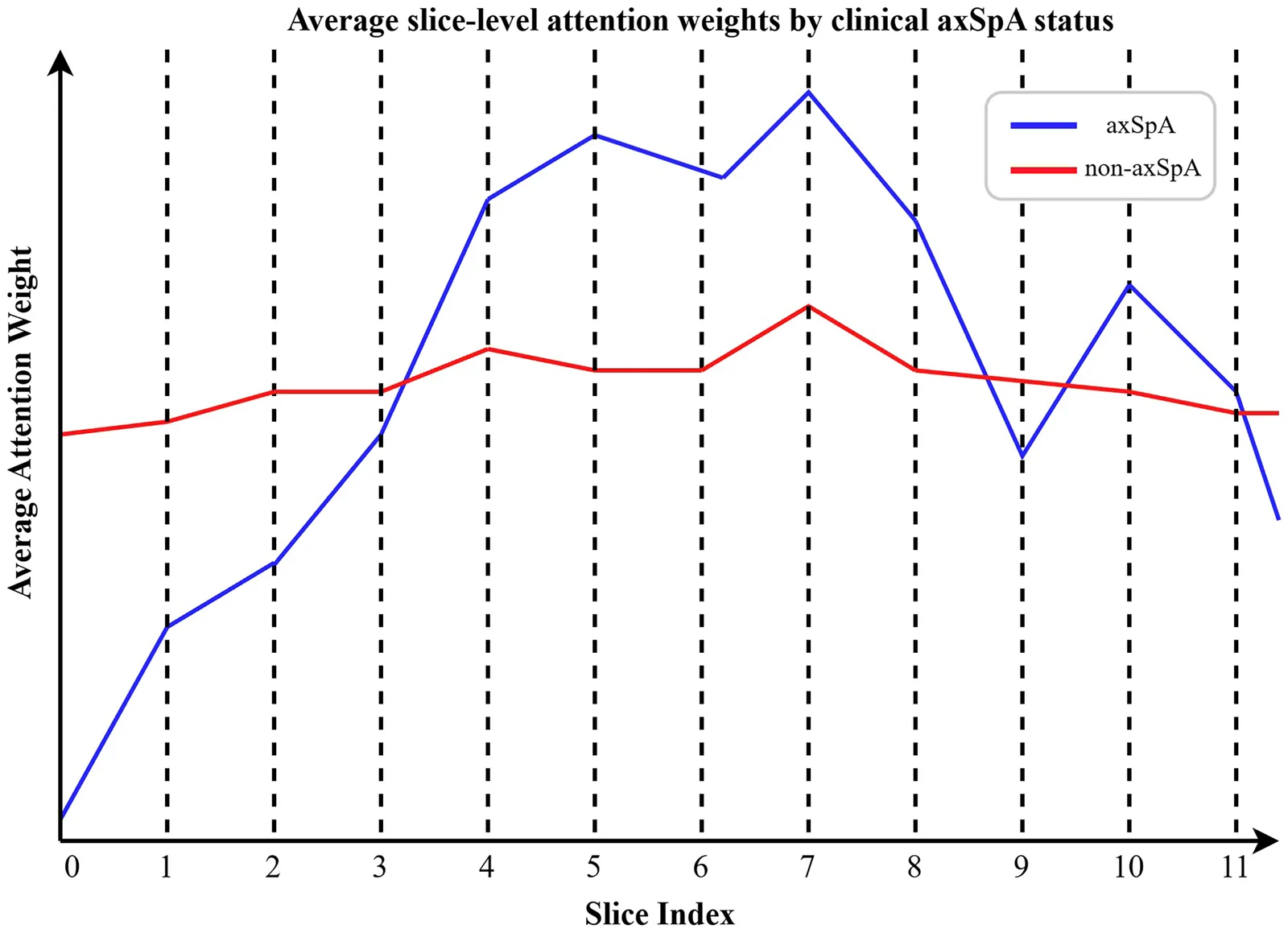
Mean slice-level attention weights according to the reference-standard clinical diagnosis. Patients with axial spondyloarthritis (axSpA) showed greater attention to the central slices, whereas patients without axSpA showed a relatively uniform distribution across slices.

### 3.3 Comparison with ASAS-based classification

On exact McNemar tests, there were no significant differences in accuracy, sensitivity, or specificity between the proposed model and either the ASAS “positive MRI” definition or the ASAS classification criteria (**Table 4**). The “positive MRI” definition had lower sensitivity (71.9% vs. 87.5% for the proposed model), whereas the ASAS classification criteria had lower specificity (76.0% vs. 84.0%).

**Table 4:** Performance of the proposed model compared with ASAS-based classification.

Hold-out test set (n=57)	Proposed model	positive MRI†		ASAS classification criteria‡	
		value	p*	value	p*
Accuracy	85.96% (49/57)	82.46% (47/57)	0.774	85.96% (49/57)	>0.999
Sensitivity	87.50% (28/32)	71.88% (23/32)	0.125	93.75% (30/32)	0.625
Specificity	84.00% (21/25)	96.00% (24/25)	0.375	76.00% (19/25)	0.754

*McNemar p-values. †ASAS definition of active sacroiliitis on MRI (‘positive MRI’), per the ASAS handbook. ‡ASAS classification criteria for axial spondyloarthritis, applying both the imaging arm and the clinical arm. ASAS, Assessment of spondyloArthritis international aociety.

### 3.4 Exploratory analysis of BME-clinical discordant cases

In an exploratory case-level analysis, 10 of the 57 patients showed discordance between the ASAS “positive MRI” assessment and the clinical diagnosis. The proposed model correctly classified 7 of these 10 cases, including 6 of 9 BME-negative/axSpA-positive cases and the single BME-positive/axSpA-negative case (**Table 5**). Given the small number of discordant cases, these findings should be interpreted descriptively rather than as evidence of subgroup performance.

**Table 5:** Exploratory case-level analysis of BME-clinical discordant cases (n=10).

Discordant patient group (*n* = 10)	Proposed model
BME-negative/axSpA-positive (*n* = 9)	6/9
BME-positive/axSpA-negative (*n* = 1)	1/1
Overall (*n* = 10)	7/10

Discordant cases are those in whom MRI-based classification by the ASAS “positive MRI” definition contradicted the final clinical diagnosis and are therefore not classified correctly by that definition. ASAS, assessment of spondyloArthritis international society; BME, bone marrow edema; axSpA, axial spondyloarthritis.

## 4 Discussion

This study demonstrated that an image-only deep learning framework integrating FS T1 and STIR SIJ MRI with slice-level binary BME predictions could predict the clinical axSpA label in patients undergoing SIJ MRI for chronic back pain. In the internal test set of 57 patients, the model achieved an AUROC of 0.9412, with no statistically significant paired differences in accuracy, sensitivity, or specificity compared with either the ASAS “positive MRI” definition or the ASAS classification criteria. In the exploratory analysis of cases discordant between the ASAS “positive MRI” assessment and the clinical diagnosis, the model correctly classified 70.0% (7/10).

Prior AI studies using SIJ MRI have mainly focused on detecting or quantifying predefined imaging findings such as BME or erosion (10, 24–26), and our own previous work likewise focused on detecting sacroiliitis itself (27). In practice, however, the object of judgment is not an individual imaging finding but the patient’s clinical classification. Previous machine-learning studies targeting axSpA diagnosis relied on clinical or electronic health record variables (17, 18), while a recent multicenter study combined clinical risk factors with SIJ MRI (19). In contrast, the present study predicted the clinical label from routinely acquired SIJ MRI alone, without clinical variables. This does not imply that axSpA can be diagnosed by MRI alone, however, and should be interpreted as indicating potential value as an image-based decision-support tool.

Integration of FS T1 was motivated by the limitations of BME-based assessment. Although the ASAS “positive MRI” definition has greatly facilitated early diagnosis, BME alone is not sufficiently specific for axSpA, and BME may not be evident at the time of imaging even in patients clinically classified as having axSpA. In contrast, structural lesions such as erosion on T1-weighted imaging have been reported to discriminate axSpA with high specificity in large prospective cohorts such as SPACE and DESIR (28, 29), and have been used as supportive diagnostic markers in practice (30). However, the present model did not explicitly identify or quantify these lesions. In the ablation analysis (**Table 3**), the full model integrating STIR, FS T1, and BME achieved a higher AUROC than the reduced-modality models. In the small exploratory subgroup of axSpA-positive patients not captured by the ASAS “positive MRI” definition, 6 of 9 were correctly classified by the proposed model. Given the small subgroup size, this observation is hypothesis-generating and should not be interpreted as evidence of subgroup performance. Accordingly, these findings suggest only that FS T1 may have provided complementary imaging information and do not establish that specific structural abnormalities were responsible for the observed performance differences.

The ASAS definition of a “positive MRI” and the ASAS classification criteria were included as contextual research comparators rather than as equivalent diagnostic tests. These approaches serve different purposes: the ASAS positive MRI definition focuses on active inflammatory lesions on MRI, whereas the ASAS classification criteria integrate imaging and clinical features and were developed for classification rather than clinical diagnosis. Moreover, the three approaches are not independent because they share imaging and/or clinical information. Accordingly, these comparisons should be interpreted as descriptive and exploratory comparisons of classification patterns within the same cohort, rather than as evidence of equivalence or superiority of the proposed model over the ASAS-based approaches. Within this context, the error profiles of the three approaches differed: the “positive MRI” definition showed a greater tendency toward false-negative classifications, whereas the ASAS classification criteria showed a greater tendency toward false-positive classifications, while the proposed model showed a relatively balanced distribution of errors in both directions. This pattern may partly reflect the composition of our cohort. The non-axSpA group did not consist of healthy controls but of patients referred for chronic back pain who underwent MRI, frequently with SpA features such as HLA-B27 positivity. This referral-stage pre-filtering may have reduced false-positive BME findings while making rule-based classification criteria more prone to overclassification. Conversely, some patients with axSpA may have had established or less active disease in which BME was absent or less conspicuous. In these patients, the additional information derived from FS T1 images may have contributed to the different classification pattern observed with the proposed model, although the specific imaging features responsible for this contribution were not identified in the present study. These cohort-specific findings may be clinically informative but should not be directly generalized to general-population or early-screening settings.

This study has several limitations. The ASAS criteria are intended for classification rather than diagnosis and were not used here as diagnostic criteria. The reference standard was the final clinical diagnosis documented by the treating rheumatologist, ascertained through longitudinal review of the documented clinical course. This diagnosis reflected the treating rheumatologist’s integrated assessment of clinical symptoms, laboratory findings, imaging findings, and subsequent disease course. Because SIJ MRI could form part of the clinical assessment used to establish the reference diagnosis, potential incorporation bias cannot be excluded and may have contributed to overestimation of model performance. Accordingly, the reported performance should not be interpreted as diagnostic accuracy against an MRI-independent disease reference standard. Rather, it reflects the extent to which an MRI-only model approximates the longitudinal clinical assessment used in routine practice, of which MRI itself may be one component. In addition, the retrospective single-center design and the small test set (n=57, with 10 discordant cases) limit generalizability and the statistical rigor of the comparative analyses; the reference standard was based on the assessment of the individual treating rheumatologist for each patient rather than a standardized multi-reader adjudication process; external validation was not performed; and the analysis was restricted to SIJ MRI, potentially underrepresenting disease burden across the spine. Because structural lesions were not explicitly annotated or quantified, the contribution of specific structural abnormalities could not be determined from the ablation or visualization analyses.

In conclusion, an image-only deep learning framework integrating FS T1, STIR, and slice-level BME information achieved an AUROC of 0.9412 for approximating the patient-level clinical assessment of axSpA in an internal hold-out test set. Exploratory findings in cases discordant between the ASAS “positive MRI” assessment and the clinical diagnosis suggest that complementary MRI information beyond BME alone may warrant further investigation. Prospective multicenter external validation is required before clinical application, and the proposed framework should be considered a decision-support tool rather than a replacement for comprehensive clinical evaluation.

## Data Availability

The original contributions presented in the study are included in the article/**Supplementary Material**. Further inquiries can be directed to the corresponding authors.
