# Supplementary material for: Artificial intelligence-based decision support for axial spondyloarthritis integrating inflammatory and structural MRI information
Source: Front Immunol. 2026 Oct 19;17:1950510. doi: 10.3389/fimmu.2026.1950510 (PMC13724556; doi:10.3389/fimmu.2026.1950510)
Supplement: Supplementary file 1 [file DataSheet1.pdf]

Supplementary Material

1 Supplementary Figures and Tables

1.1 Supplementary Tables

Supplementary Table S1. Summary of dataset splits across the hierarchical development stages. A strict patient-wise partitioning strategy was implemented to ensure that no subject appeared in multiple subsets, thereby preventing data leakage. Stratified sampling was applied to preserve the prevalence of positive cases across the training, validation, and test sets.

Stage	Task	Level	Total	Train (P/N)	Val (P/N)	Test (P/N)
Stage 1	SIJ Localization	Slice	2,328	1,479	370	479
	BME Classification	ROI	4,626	2,958 (486/2,472)	740 (121/619)	928 (226/702)
Stage 2	axSpA Classification	Patient	291	187 (105/82)	47 (26/21)	57 (32/25)

Note. The same patient-wise split was used across all tasks. Validation sets were used for checkpoint selection and early stopping, and test sets only for final evaluation. P/N, positive/negative ROIs for BME and patients for axSpA; SIJ, sacroiliac joint; BME, bone marrow edema; ROI, region of interest; axSpA, axial spondyloarthritis; Val, validation.

Supplementary Table S2. Training configurations and hyperparameters for the proposed two-stage deep learning framework.

Configuration	Parameter / Metric	Stage 1		Stage 2
		SIJ Localization	BME Classification	axSpA Classification
**Network Architecture**	Backbone / Model	YOLOv12x	ConvNeXt-Large	EfficientNet-B4 + MLP + Self-Attention
**Input Specifications**	Input Resolution	512×512	512×512	256×256 (12 slices per patient)
	Input Channels	1 (STIR, Grayscale)	3 (STIR, repeated 3 times)	FS T1 + STIR (3 channels each) + binary BME predictions
	Optimizer	SGD	AdamW	AdamW
	Base Learning Rate	1×10^{-3}	5×10^{-6}	3×10^{-5}
	Weight Decay	1×10^{-4}	1×10^{-5}	1×10^{-4}
	Learning Rate Scheduler	Cosine Annealing	Cosine Annealing Warm Restarts	Cosine Annealing Warm Restarts
	Loss Function	CIoU + DFL + BCE Loss	Hybrid (Focal Tversky + BCE)	Weighted BCE
**Hyperparameters**	Batch Size	16	4	7 (patients)
	Classification Threshold	-	0.5	0.5
	Total Epochs	100	300	200
	Early Stopping Patience	15 epochs	20 epochs	20 epochs
**Data Augmentation**	Geometric Transforms	Resize (512, 512)	Random Rotation (±10°), Horizontal Flip	Random Flip, Rotation (±5°), Affine, Perspective, Motion, Anisotropy
	Intensity, Color Transforms	Intensity, Color Transforms	Histogram Matching	Histogram Matching, CLAHE, Color Jitter, Bias Field, Gamma

Note. SIJ, sacroiliac joint; BME, bone marrow edema; SGD, stochastic gradient descent; CIoU, complete intersection-over-union; DFL, distribution focal loss; BCE, binary cross-entropy; CLAHE, contrast-limited adaptive histogram equalization. axSpA, axial spondyloarthritis; FS T1, fat-suppressed T1-weighted; STIR, short tau inversion recovery; MLP, multilayer perceptron; NA, not applicable.

Supplementary Table S3. MRI scanner platforms and acquisition parameters in the final study cohort (n=291).

Category	Parameter	FS T1	STIR
**Acquisition**	Field strength	3.0 T	3.0 T
	Acquisition type	2D oblique coronal	2D oblique coronal
**Timing**	Repetition time, ms	573 (450–781)	4,978 (3,884–6,692)
	Echo time, ms	14.8 (9.0–24.3)	56.8 (36.0–97.5)
	Inversion time, ms	NA	230 (200–250)
**Readout**	Echo train length	3 (2–7)	15 (11–20)
	Number of signal averages	1 (1–3)	2 (1–3)
	Excitation flip angle	90°	90°
	Pixel bandwidth, Hz/pixel	230 (195–388)	260 (209–339)
**Geometry**	Slice thickness, mm	4.0	4.0
	Slice spacing, mm	4.4	4.4
	In-plane resolution, mm	0.446 (0.244–0.694)	0.488 (0.279–0.744)
	Field of view, mm	250 (200–250)	250 (200–250)
**Coverage**	Source slices per patient	8 (1–13)	8 (4–13)

Note. Values are presented as median (range) unless otherwise indicated. FS T1-TSE, fat-suppressed T1-weighted turbo spin echo; STIR-TSE, short-tau inversion-recovery T2-weighted turbo spin echo; NA, not applicable. MRI examinations were acquired using MAGNETOM Skyra (Siemens Healthineers; n=143), Ingenia or Ingenia CX (Philips Healthcare; n=121), and Achieva (Philips Healthcare; n=27). The DICOM FlipAngle field was vendor-dependent: Philips systems reported the 90° excitation angle, whereas Siemens systems reported refocusing flip angles of 120°–150°.

Supplementary Figures

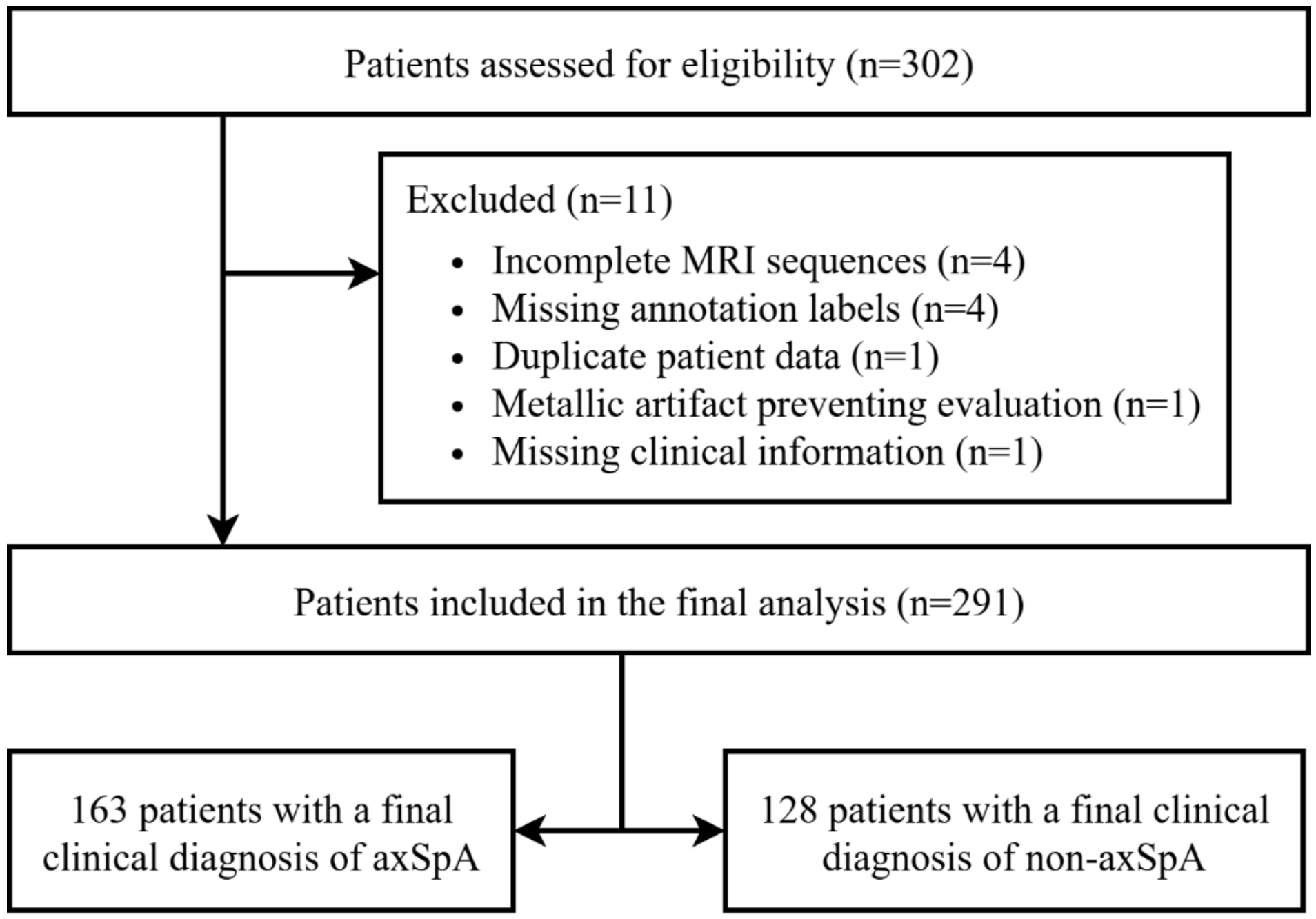

Supplementary Figure S1. Participant selection flowchart for the STIR and FS T1 SIJ MRI dataset, showing the final cohort composition according to the final clinical diagnosis.

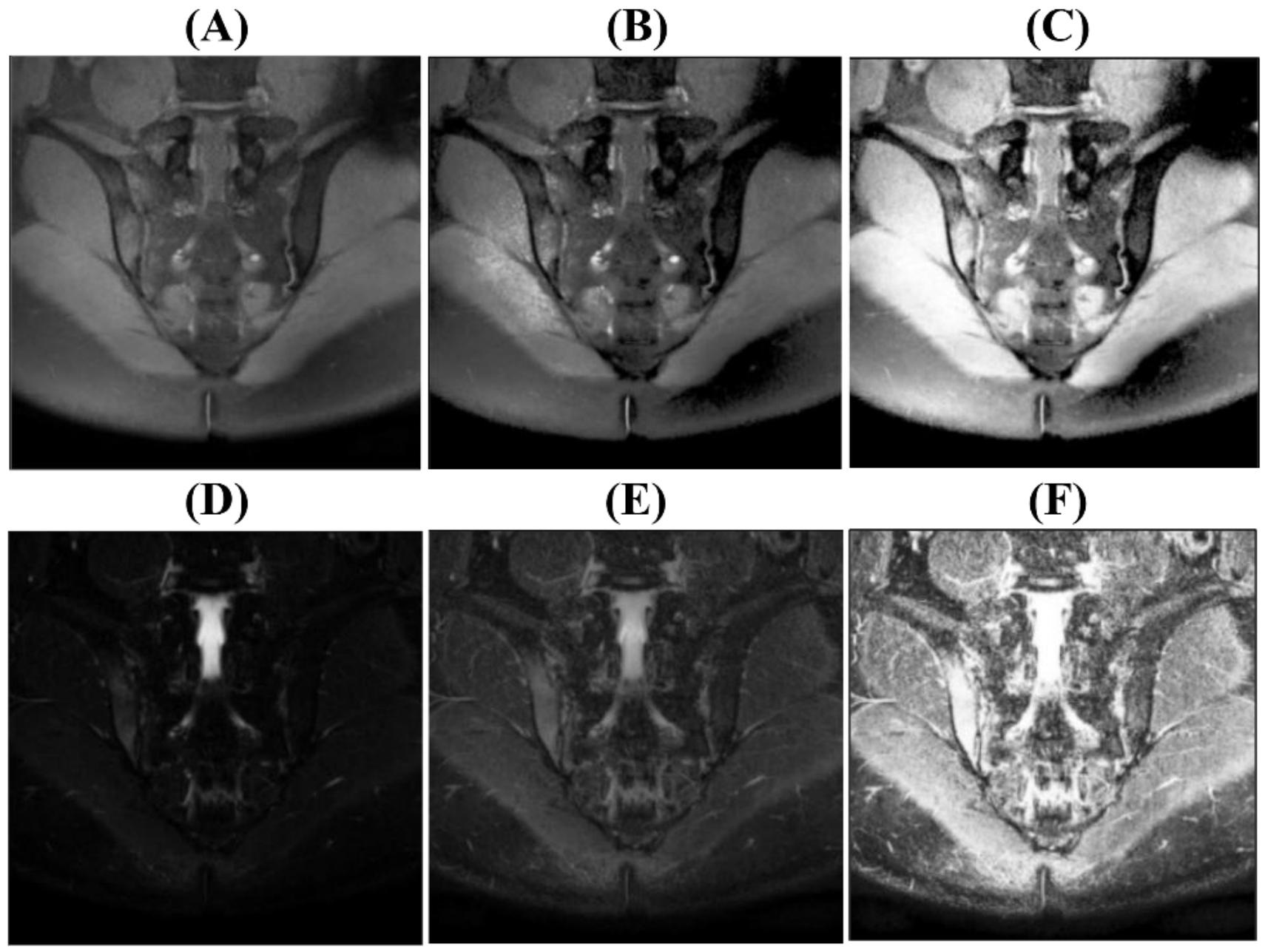

Supplementary Figure S2. Standardized preprocessing pipeline for multivendor sacroiliac joint magnetic resonance imaging. Panels A–C show fat-suppressed T1-weighted images, and panels D–F show short tau inversion recovery images. Panels A and D show the original images; panels B and E show the images after histogram matching to a radiologist-selected reference; and panels C and F show the images after contrast-limited adaptive histogram equalization.

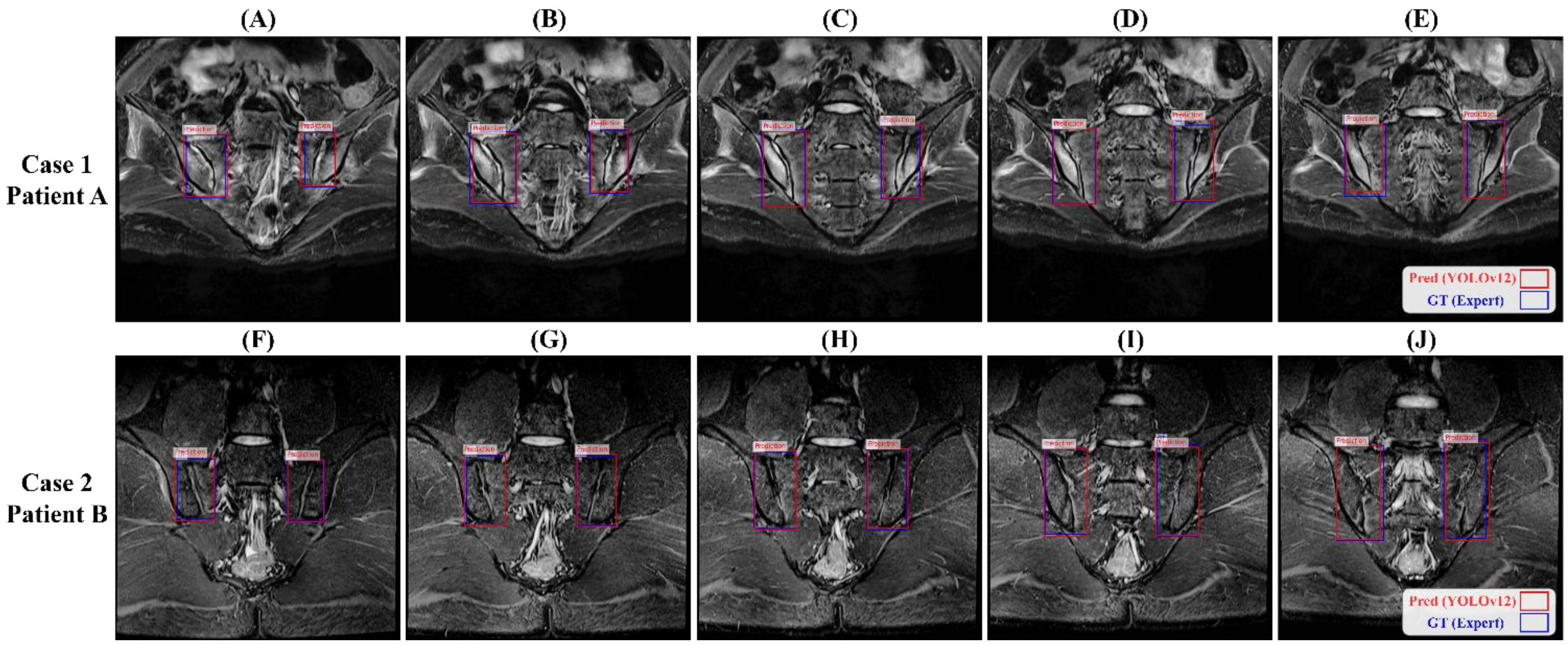

Supplementary Figure S3. Sacroiliac joint localization on coronal STIR MRI. Case 1 (Patient A, A–E) and Case 2 (Patient B, F–J) show five sequential slices each; ground-truth bounding boxes are shown in blue and YOLOv12 predictions in red.
